# TSEA-DB: a trait–tissue association map for human complex traits and diseases

**DOI:** 10.1093/nar/gkz957

**Published:** 2019-11-04

**Authors:** Peilin Jia, Yulin Dai, Ruifeng Hu, Guangsheng Pei, Astrid Marilyn Manuel, Zhongming Zhao

**Affiliations:** 1 Center for Precision Health, School of Biomedical Informatics, The University of Texas Health Science Center at Houston, Houston, TX 77030, USA; 2 Human Genetics Center, School of Public Health, The University of Texas Health Science Center at Houston, Houston, TX 77030, USA; 3 MD Anderson Cancer Center UTHealth Graduate School of Biomedical Sciences, Houston, TX 77030, USA; 4 Department of Biomedical Informatics, Vanderbilt University Medical Center, Nashville, TN 37203, USA

## Abstract

Assessing the causal tissues of human traits and diseases is important for better interpreting trait-associated genetic variants, understanding disease etiology, and improving treatment strategies. Here, we present a reference database for trait-associated tissue specificity based on genome-wide association study (GWAS) results, named Tissue-Specific Enrichment Analysis DataBase (TSEA-DB, available at https://bioinfo.uth.edu/TSEADB/). We collected GWAS summary statistics data for a wide range of human traits and diseases followed by rigorous quality control. The current version of TSEA-DB includes 4423 data sets from the UK Biobank (UKBB) and 596 from other resources (GWAS Catalog and literature mining), totaling 5019 unique GWAS data sets and 15 770 trait-associated gene sets. TSEA-DB aims to provide reference tissue(s) enriched with the genes from GWAS. To this end, we systematically performed a tissue-specific enrichment analysis using our recently developed tool *deTS* and gene expression profiles from two reference tissue panels: the GTEx panel (47 tissues) and the ENCODE panel (44 tissues). The comprehensive trait–tissue association results can be easily accessed, searched, visualized, analyzed, and compared across the studies and traits through our web site. TSEA-DB represents one of the many timely and comprehensive approaches in exploring human trait–tissue association.

## INTRODUCTION

The past decade has witnessed a dramatic growth of genome-wide association studies (GWAS) to investigate the susceptibility of genetic variants that are associated with human traits and diseases (1,2). However, the interpretation of trait-associated genetic variants currently remains an open challenge. One powerful approach is to study the potential roles of genetic variants through expression quantitative trait loci (eQTL) analyses (3). One example is the rapid emergence of transcriptome-wide association studies (TWAS) which link the DNA variation with gene expression for better searching reliable genetic association signals. However, because genetic regulation of gene expression is highly tissue specific, a critical step is to identify the tissue and cell-type context when interpreting genetic variants. Several methods have been reported to identify causal or relevant tissues to traits (4–6). In some methods, eQTL are implemented to identify the tissues in which the trait-associated loci are most likely functional (6), whereas in other methods gene expression profiles are used to conduct tissue-specific enrichment analysis (TSEA) (4). We recently developed a software package, *deTS*, to decode tissue specificity based on reference gene expression panels and demonstrated the robustness of the methods (chi-square test for a list of genes and *t*-test for transcriptomes) in 26 traits and 14 cancer types (7).

Tissue specificity of gene expression has been widely investigated in many studies, ranging from Mendelian disorders (8,9) to complex diseases (10,11), from germline variants (5) to somatic mutations (12,13), and from basic biological processes (14) to drug response (15,16). Importantly, decoding the tissue specificity of GWAS-implied loci (e.g. genes) has many important applications. First, it provides a reference of tissues in which trait-associated genes likely act (17). While a number of traits are known for their causal tissues (e.g. cancer (18), diabetes), many other traits remain elusive. TSEA of GWAS data enables an unbiased investigation of tissues in which GWAS-implied genes are enriched. Second, identification of the trait–tissue associations will provide insights into trait-trait relationships and possibly disease comorbidity. Traits that are associated with the same tissue likely share genes or pathways in their underlying mechanisms (19). Third, trait-associated tissues serve as an attractive implication for identification and interpretation of causal variants. Many computational methods require analyses to be conducted in a disease-relevant tissue context. For example, TWAS have prediction models that are trained for each tissue separately (20) and colocalization tests are conducted in a tissue-specific manner (21). Therefore, an exploration of trait-associated tissues and a trait–tissue association map will provide a valuable reference for these studies.

In this work, we conducted TSEA using our *deTS* package for >5000 GWAS summary statistics data sets and constructed the database TSEA-DB to present these results to the public. The GWAS data were collected from several major sources and literature mining. We built a pipeline to perform strict quality control of the data. *deTS* was applied to each qualified GWAS data set using two reference panels, one from the Genotype-Tissue Expression (GTEx) project (47 tissues) (22) and the other from the Encyclopedia of DNA Elements (ENCODE) project (44 tissues) (23). The design of multiple layers of trait-associated genes (TAGs) from GWAS data with different thresholds allows users the flexibility to explore the data with specific interests. Finally, all results are publicly available to the users with specific data freezes (update with the continuous growth of GWAS), serving as a reference to trait–tissue association map.

## DATA COLLECTION, ANALYSIS AND INTEGRATION

### GWAS summary statistics

#### Data collection

The GWAS summary statistics data were collected and processed from the following sources: major multi-trait studies (21,24,25), GWAS Catalog (26), the UK Biobank (UKBB) resource (http://www.nealelab.is/uk-biobank) (2), and extensive literature mining. Among them, we named the data sets from the Multi-Trait Collection as the MTC panel. This panel, which comprised of the data from several publications (21,24,25), is fixed and will not be updated in future. We named the union of data sets from GWAS Catalog and from literature mining as the Expanded Trait Collection (ETC). The ETC panel is under active curation and will be updated routinely in future. Both Multi-Trait and Expanded Trait panels are also collectively referred to the non-UKBB panel in our database. The UKBB panel, which was originally preprocessed by http://www.nealelab.is/uk-biobank and was released in 1 August 2018 (referred originally as ‘GWAS round 2’), represents the largest resource of GWAS data sets for traits/diseases. We downloaded the data sets using all samples including both men and women and will update the data by closely following the original project. We also explored other resources of UKBB data, such as Gene ATLAS (27). By comparing the covariates and genomic inflammation factor, we found that the release by http://www.nealelab.is/uk-biobank had stronger quality controls, more comprehensive covariates (20 principal components, age, age^2^, sex, age × sex and age^2^ × sex), relatively less inflation, and more phenotypes. Accordingly, we used the data processed by http://www.nealelab.is/uk-biobank.

#### Quality control (QC) of GWAS data

We applied the following filtering criteria for the non-UKBB panel whereas the UKBB panel has been under comprehensive quality control by the original consortium. First, we included only GWAS datasets that were conducted using samples of European ancestry. We excluded those meta-analyses covering trans-ethnic cohorts because there was no appropriate linkage disequilibrium (LD) panel for calculation of gene-based *P*-values (see below). Second, only the studies having >100,000 analyzed genetic variants [mainly single nucleotide polymorphisms (SNPs)] with genome-wide coverage were included. Third, we removed the studies having incomplete information, e.g. those with missing SNP IDs (NCBI rsIDs). Lastly, we calculated the genomic inflation factor λ and removed studies with large λ values (i.e. λ > 1.5). This step excluded data files where only partial list of genetic variants were provided (e.g. those with *P* < 0.05). Note that the λ cutoff value 1.5 was relatively large for the typical GWAS analyses (typically from 1.0 to 1.1). We chose this cutoff because in some studies, especially those meta-analyses, their λ values were quite high (e.g. 119 data sets had λ between 1.2 and 1.3), likely due to the dense genotyping after imputation. For example, when many SNPs share a strong LD with the causal locus, the λ value could become high.

#### Calculation of gene-based *P*-value

We used the Pathway scoring algorithm (Pascal) (28) to calculate gene-based *P*-values based on the GWAS summary statistics. SNPs with minor allele frequency (MAF) ≤ 0.05 were filtered out. For each gene, we included the SNPs that were located in the gene body or 50kb upstream/downstream of the gene body. Pascal employs LD information in the reference panel to estimate the SNP–SNP relationship. In our application, we chose the European panel from the 1000 Genomes Project (1KGP) (29) as the reference panel. SNPs in the Major Histocompatibility Complex (MHC) region on chromosome 6 or in sex chromosomes were excluded (30). Hence, in the following analyses, only genes on autosomal chromosomes were included. For the raw Pascal results, we selected genes that were successfully analyzed by Pascal and excluded the poorly genotyped genes. After this step, we further excluded several GWAS data sets because Pascal failed to generate the appropriate results or the number of successful genes was <18 000. To further explore the distribution of association results, we drew two Manhattan plots for each GWAS set using SNPs and genes after QC, respectively, and made them available through our database website. To keep consistent, only autosomal chromosomes were included on the Manhattan plots.

#### Exclusion of duplicated data sets

Using the Pascal gene-based *P*-values, we calculated a pair-wise correlation coefficient to identify potential duplicated data sets from different sources. If two data sets had a Pearson Correlation Coefficient (PCC) > 0.99, we randomly chose one to include in our database. It is worth noting that this step is to exclude duplicated rather than similar data sets. For example, even though two data sets ‘Sleep duration’ (https://bioinfo.uth.edu/TSEADB/showTraitDeTS.do?gwasid=4620) and ‘Sleep duration adjusted for BMI’ (https://bioinfo.uth.edu/TSEADB/showTraitDeTS.do?gwasid=4619) had a PCC = 0.98, both were included because they represent two unique data sets. Our goal is to provide as comprehensive results as possible of the GWAS summary statistics and only excludes the redundant data sets.

### Human tissue transcriptome data and *deTS*

We collected two reference panels for tissue expression profiling: the GTEx panel and the ENCODE panel, the two widely-used expression panels currently available. The original GTEx data (version 7) had expression profiles for 53 tissues (22). We selected 47 tissues by requiring sample size at least 30. A *t*-score was calculated for each gene to measure its tissue specificity for each tissue after adjusting for covariates (7). For the ENCODE data, we collected 44 tissues, each of which had at least two independent samples. Due to the small sample size, we calculated a *z*-score for each gene in each tissue to better estimate the tissue specificity. Through our manual examination of the location and function of all tissues, we determined 20 shared or similar tissues between the two tissue panels and a total of 32 unique tissue groups (Supplementary Table S1). Both panels were made available in our *deTS* package and TSEA-DB.

### Definition of trait-associated gene (TAG) set

For each GWAS data set, we defined five TAG sets by using different thresholds for the gene-based *P*-value from Pascal: *P* < 0.05, *P* < 0.01, *P* < 1 × 10^−3^, *P* < 1 × 10^−4^, and *P* < 1 × 10^−5^. Among them, if a TAG set had >3000 genes or <20 genes, the set would not be analyzed by *deTS* due to too large or too small size in statistical analysis. The qualified TAG sets are labeled in bold on the trait page. Having multiple TAG sets for each GWA study allows users to explore the enriched tissues at different scales and increases the chance to identify the appropriate tissues, because each trait/disease may be related with multiple tissues with different extent. As a final QC step, for each GWA study, we required at least one TAG set passing the criteria so that *deTS* could be subsequently applied. GWAS data sets without any qualified TAG set were thus excluded. After these quality control steps, we obtained 435 GWAS summary statistics data sets in the Multi-Trait Collection panel, 161 in the Expanded Trait Collection panel, and 4423 in the UKBB panel, totaling 5019 GWAS data sets and 15 770 qualified TAG sets. There were 439, 613, 3206, 744 and 17 GWAS data sets that had five, four, three, two and one qualified TAG set, respectively.

### Application of *deTS* and TSEA

For each TAG set, we applied *deTS* using the default chi-square test for the association test. Specifically, for each tissue, we defined the top 5% genes, ranked by tissue *t*-scores (or *z*-scores), as the tissue-specific genes. The two groups of genes, i.e. tissue-specific genes and TAGs, were compared to build a 2 × 2 contingency table. Chi-square test was implemented in R and the one-sided *P*-value for an overrepresentation of shared genes was reported.

For each GWAS data set, we selected three tissues with the most significance and performed Gene Set Enrichment Analysis (GSEA) for further evaluation using the *fgsea* package in R (31). We modified the default parameters of the plot function of *fgsea* to label genes with their Pascal *P*-values and thus, it could be visualized how the genes with association *P*-values were enriched in each trait-associated tissue. For each tissue and TAG pair, we applied 10 000 permutations and calculated the normalized enrichment score (NES). A higher NES score indicates that the TAGs are more specifically expressed in the examined tissue. Both the permutation *P*-value and NES are reported for each examined tissue.

## APPLICATIONS OF THE TRAIT-TISSUE ASSOCIATION MAP

### Traits with enriched tissues

**Figure 1.**
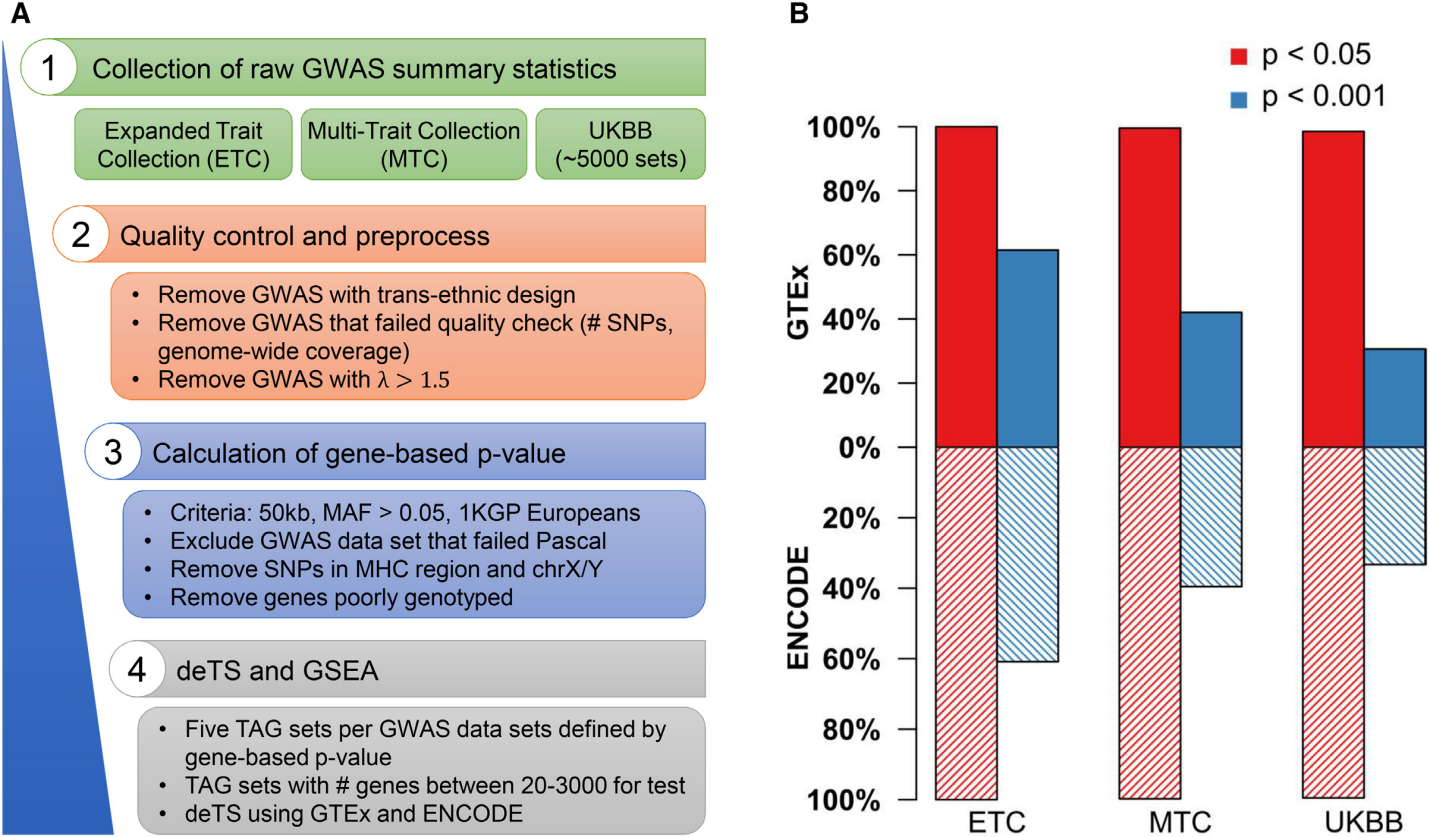
Data curation and quality control. (**A**) A pipeline of data quality control process. UKBB: UK Biobank. 1KGP: The 1000 Genomes Project. TAG: trait-associated gene. (**B**) Comparison of traits with enriched tissues in the GTEx panel and the ENCODE panel. The red and blue bars are plotted using trait–tissue associations defined using *deTS P*-value < 0.05 and *deTS P*-value < 0.001, respectively.

The current database had 15 770 qualified TAG sets from the 5019 GWAS data sets. By applying *deTS P*-value < 0.05, we identified 13 247 (84.0%) qualified trait-TAG sets with significant trait–tissue associations, covering a total of 4952 traits (Figure 1). These results included 161 (100%) Expanded Trait Collection data sets, 433 (99.5%) Multi-Trait Collection traits and 4358 (98.5%) UKBB data sets. For the GTEx panel, we further used the *P*-value cutoff 0.001 (0.05/47, Bonferroni correction) to define significant trait–tissue associations. Although this may be considered an arbitrary value, using all trait × tissue pairs as the number of tests would be too strict. By setting threshold of the trait–tissue association *P*-value < 0.001, we found 19.0% (3000/15 770) trait-TAG sets having at least one significantly enriched tissue, covering 99 (61.5%) Expanded Trait Collection data sets, 183 (42.1%) Multi-Trait Collection data sets, and 1355 (30.6%) UKBB data sets. For the ENCODE panel, by requiring the trait–tissue association *P*-value < 0.05, we found that 87.8% (13 841/15 770) trait-TAG sets had significant trait–tissue associations. Applying the same cutoff of 0.001 (0.05/44, Bonferroni correction), there were 98 Expanded Trait Collection data sets, 172 Multi-Trait Collection data sets and 1473 UKBB data sets having at least one significant tissue. The two panels shared 986 GWAS data sets (19.6% of 5019 data sets) with significant associations (1637 from GTEx and 1743 from ENCODE) at *deTS P* < 0.001.

### Trait-enriched tissues

We also collected all traits whose TAGs were enriched in each tissue. For each GTEx tissue, there were on average 151 (median: 127 and range: 6–712) TAG sets from 95 (median: 79 and range: 6–289) GWAS data sets using *deTS P* < 0.001. The tissues having the largest number of enriched GWAS data sets were spleen (289 GWAS data sets), lung (234), whole blood (227), and brain–frontal cortex (BA9) (159). These results were similarly replicated in the ENCODE tissue: the tissues having the largest number of enriched GWAS data sets was spleen (310), parietal lobe (232), upper lobe of left lung (210) and suprapubic skin (176). This is very promising because ENCODE tissues had much smaller sample sizes. This high level of consistency indicated that our panels and the *deTS* results were reproducible.

### Asthma as an example

**Figure 2.**
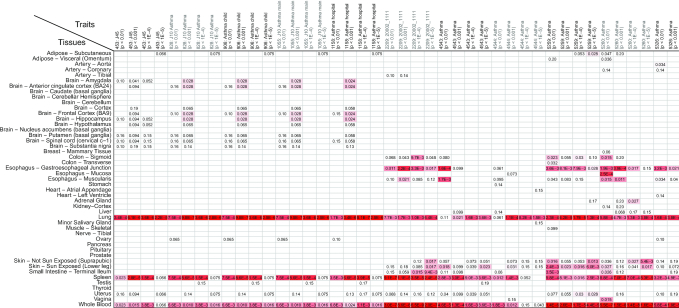
An example to compare asthma GWAS from 11 data sets and 44 TAG sets. Due to space limitation, we excluded the TAG sets with Pascal *P*-value < 0.05.

Thirty-two GWAS data sets were found in TSEA-DB when searching using the keyword ‘asthma’. After a manual check, we selected 11 data sets that had >1000 cases in specific asthma studies. We compared the *deTS* results from all 11 GWAS data sets and examined the enriched tissues. As shown in Figure 2, three tissues (lung, spleen, and whole blood) were overall consistently enriched in the GTEx panel. Among them, lung and spleen were the shared tissues between the GTEx and ENCODE panels, while whole blood is only detected by the GTEx panel. By examining the enrichment results using the ENCODE panel, we found that both lung and spleen were also enriched in the ENCODE panel; this demonstrated our resource is useful because the GWAS data sets were from independent cohorts of asthma, indicating that the results were reproducible not only across the studies but also across our reference gene expression panels. Asthma is a heterogeneous disorder characterized by chronic inflammation of the respiratory airways, with conditions such as a patient's airways being inflamed, narrow and swell and producing extra mucus. Our results of both lung (respiratory system) and spleen (where white blood cells are stored) support that the genetic signals have the direct link (or potentially causal) to the specific disease.

## DESCRIPTION OF THE WEBSITE AND TOOLS

### General functions: browse, search and visualization

#### Browse of traits

Users can browse traits (GWAS), tissues, and trait–tissue associations through TSEA-DB website. It provides functions to browse traits by panels (the ETC panel, MTC panel, UKBB panel, or non-UKBB panel) or by tissues. Notably, for the UKBB panel, there are 632 GWAS data sets that are annotated using the main ICD10 code. We mapped these traits using the 2019 International Classification of Diseases, 10th Revision, Clinical Modification (ICD-10-CM) diagnosis codes (https://www.icd10data.com/ICD10CM/Codes) with levels 1 and 2. These data sets are provided as a special subset of the UKBB panel and can be browsed using the *Dataset* function. Users can also search traits or tissues by keywords or using combinations of conditions.

#### Trait page

**Figure 3.**
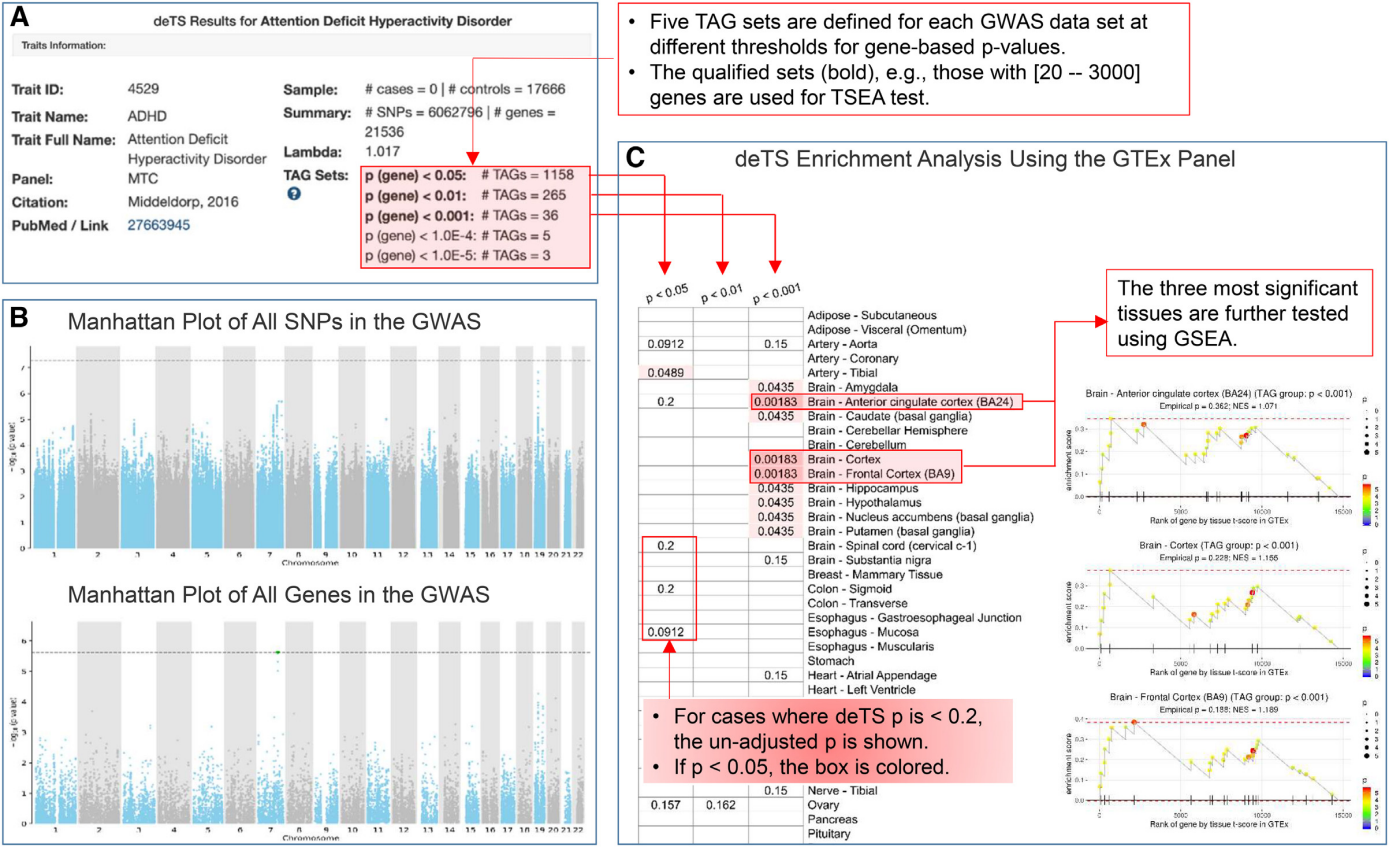
Illustration of the trait page using the GWAS data set for attention deficit hyperactivity disorder (ADHD). (**A**) Basic information of the corresponding GWAS data set. (**B**) Manhattan plots using all SNPs (top) and all genes (bottom) of the GWAS data set. (**C**) deTS results using the GTEx panel. Boxes in red are annotations.

The trait page has abundant information (Figure 3). It can be visited by the *search* or *browse* functions. The trait page starts with basic information of a GWAS data set, including full trait name, PubMed link, sample size, number of SNPs and genes after quality control, λ value and the qualified TAG sets. Next, the page presents Manhattan plots for all SNPs and for all genes to show the overall association distributions. As aforementioned, sex chromosomes are excluded. The top and bottom display of the two plots allows users to explore and determine if the gene-based *P*-values are consistent with the original GWAS association results. The *deTS* results are subsequently presented for the GTEx panel and the ENCODE panel, respectively. For each panel, the results from *deTS* for all qualified TAG sets are shown as a heatmap, with the unadjusted *P*-values presented and highlighted using gradient colors. The figure can be downloaded as a SVG plot. It further selects the top three tissues with the most significant enrichment results and validates using GSEA. Specifically, for a tissue in examination, all genes with GTEx *t*-values are pooled. Genes are ranked with a decreasing *t*-value, indicating decreasing tissue specificity. The TAG set with the smallest *deTS P*-value is selected and considered as the gene set. We modify the original plot function to highlight TAG genes in the GSEA plot, where the color theme is proportional to their Pascal *P*-values. Both the permutation *P*-value and NES are shown in the plot. For the ENCODE panel, the layout of results is similar to the GTEx panel and GSEA is applied with genes ranked by decreasing z-scores.

#### Tissue page

We allow users to search or browse by tissue. For each tissue, the tissue page presents a dynamic trait–tissue association network including traits that are nominally significantly associated with the tissue. Due to space limitation, only traits from the non-UKBB panel are plotted. The network view is implemented by the Cytoscape plugin (32). Users can drag and zoom in or out to better view the network. We take Adipose–Visceral (Omentum) as an example (Supplementary Figure S1). This is a tissue from the GTEx panel with 363 samples. At nominal *P*-value < 0.05, there are 189 TAG sets from 105 non-UKBB GWAS data sets significantly associated with this tissue. These traits are mainly adiponectin, waist hip ratio (WHR), blood lipids, and fasting insulin, among others. Similarly, for the ENCODE tissue Omental Fat Pad, which is a counterpart tissue of the GTEx Adipose – Visceral (Omentum), a total of 146 TAG sets for 82 GWAS data sets are associated with the ENCODE Omental Fat Pad. Forty-five GWAS data sets (42.9% of those associated with GTEx Adipose–Viseral Omentum and 54.9% of those associated with Omental Fat Pad from ENCODE) are overlapped with the two panels, which is significantly higher than expected (*P* < 2.2 × 10^−16^, Fisher's Exact Test).

#### Documentation page

All data collection, preprocessing and summary are presented in the documentation page (https://bioinfo.uth.edu/TSEADB/html/document.jsp). Illustration on how to use the *search*, *browse*, and *compare* functions as well as explanation of each page is available too.

### The function to compare multiple traits

**Figure 4.**
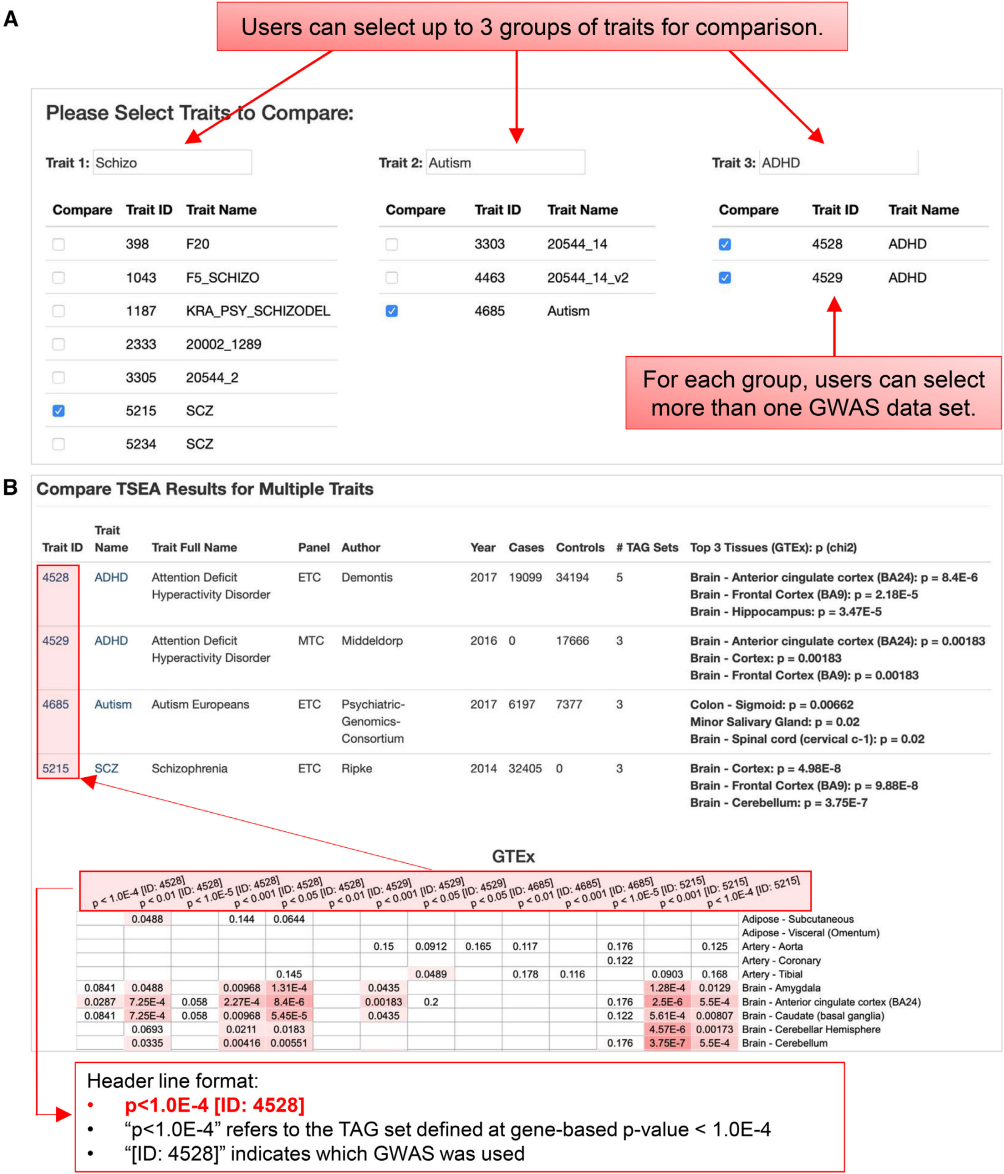
Illustration of the *compare* function with the input page and the result page. (**A**) Screenshot of the input page to select GWAS data sets for comparison. (**B**) Demonstration and illustration of the *deTS* results for multiple data sets.

The *compare* function allows users to compare multiple traits. This is an important task on the study of multi-traits or for multiple studies of the same trait. The *compare* function can be evoked from the search results page of traits or from the *compare* function directly. For example, if one searches using ‘asthma’, all GWAS data sets that have asthma in the trait name or trait full name will be listed on the search results page. This list may include GWAS conducted by different groups or with related phenotypes, e.g. asthmaticus, asthma with adult onset and asthma with childhood onset. A link to compare multiple GWAS data sets is available at the bottom of the page, which leads to a new page where users can select multiple data sets of interests for comparison of the *deTS* results. Alternatively, in the *compare* function page, we provide text boxes to allow users to select traits with different keywords. To present the *deTS* results concisely in one page, we allow up to 3 traits and up to 10 data sets for comparison at the same time. Figure 4 shows an example for multiple GWAS data sets of three psychiatric disorders: schizophrenia, autism, and attention deficit hyperactivity disorder (ADHD). These three disorders have been found with some shared genetic variants, but also many results are different among them. In this case, we selected four GWAS data sets, including one for schizophrenia, one for autism, and two for ADHD (one considering ADHD as a binary phenotype and the other as a continuous phenotype measured by a scale). Both ADHD data sets and the schizophrenia data set show an enrichment in brain regions, also evident in the ENCODE panel. However, the GWAS-implied genes for autism do not show clear enrichment pattern in either GTEx or ENCODE. This is not surprising because the original autism GWAS data set we selected does not have significant loci at the genome-wide significance level 5 × 10^−8^ (33). Collectively, such analyses provide a convenient tool for studies across different phenotypes to compare shared tissues or disease comorbidity.

## DATABASE DESIGN AND UPDATES

TSEA-DB is developed using JavaServer Pages (JSP) and JavaScript (JS). All data are stored in a local MySQL database. The web site is hosted by an Apache TomCat server and has been maintained by the university IT staff. The design and the structure of the database allows us to easily expand and add new data. The pipeline to run quality check, plot the Manhattan figures, run Pascal, and conduct *deTS* has been readily implemented using customized scripts. This allows us to efficiently process any new data sets when they are available and update regularly. Note that all the results reported in this work are based on the ‘freeze’ set of data released on 6 August 2019 so that the users can track their results.

## CONCLUDING REMARKS AND FUTURE DEVELOPMENT

Decoding disease-relevant tissues is of utmost importance to understand disease etiology. Many data types have been explored for this task, such as histone marks, expression profile, and eQTL annotations, along with various methods. In this work, we employed the raw GWAS signals and tissue gene expression to infer trait–tissue associations at large-scale. In TSEA-DB, we have observed many enrichment associations that could recapture known biological mechanisms, as well as provided new insights. For example, Crohn's disease is defined as a chronic inflammatory bowel disease. While enrichment is expected to be observed in the spleen and small intestine, both involved in Crohn's disease, it is also surprising to observe enrichment in lung. Importantly, with our comprehensive collection of GWAS data sets from various studies with many cohorts and two independent expression panels, we could validate and compare the trait–tissue associations across studies. Indeed, for the three non-UKBB GWAS data sets for Crohn's disease, we found lung was enriched in all three data sets in the GTEx panel and upper lobe of left lung in the ENCODE panel. Nevertheless, our method still could suffer from the underpowered GWAS. Users could obtain the insight of this information from the Manhattan plot and sample size in each trait page. Collectively, TSEA-DB presents a comprehensive and complementary resource to the current field.

Currently, TSEA-DB deposits GWAS data sets that were analyzed using both males and females. In future, we will also include the UKBB GWAS data sets that are based on males only or females only, and will use new UKBB version too. The gene expression profile for the ENCODE panel will be updated when new data are available, including both new samples and new tissues. GTEx v8 was released in August 2019. We will process the data, re-analyze it, and update our TSEA-DB. In addition, we will expand to include GWAS summary statistics of other ethnicity ancestries, such as those conducted using individuals of African, Latin American, and/or Asian ancestry. Another important resource is cell type expression. There are a few ongoing large-scale consortium projects such as Human Cell Atlas (34). When such data is available, we will incorporate into our *deTS* tool and TSEA-DB database. In addition to complex traits and diseases, tissue-specificity can be insightful to understand many other biological problems, such as pathway activities, drug responses, and protein-protein interactions, to name a few. We will continue our efforts to improve the methods in *deTS* and extend our current methods for TSEA of new data types. In summary, while there have been many methods for trait–tissue associations, TSEA-DB provides a complement solution with user-friendly design and comprehensive analyses.

## Supplementary Material

gkz957_Supplemental_Files
