# Supplementary material for: TSEA-DB: a trait–tissue association map for human complex traits and diseases
Source: Nucleic Acids Res. 2019 Nov 4;48(D1):D1022–30. doi: 10.1093/nar/gkz957 (PMC7145616; doi:10.1093/nar/gkz957)
Supplement: gkz957_Supplemental_Files [file gkz957_supplemental_files.zip › Table S1.docx]

**Table S1**. Summary of tissue information.

|  |  | **GTEx** | | | **ENCODE** | | |
| --- | --- | --- | --- | --- | --- | --- | --- |
| **Tissue  Group** |  | **Tissue ID** | **Tissue Name** | **Sample** | **Tissue ID** | **Tissue Name** | **Sample** |
| **G1*** | Adipose - Subcutaneous | T1 | Adipose - Subcutaneous | 485 | E1 | Subcutaneous Adipose Tissue | 4 |
| **G1** | Adipose - Visceral (Omentum) | T2 | Adipose - Visceral (Omentum) | 363 | - | - | 0 |
| **G1** | Omental Fat Pad | - | - | 0 | E2 | Omental Fat Pad | 4 |
| G2 | Artery - Aorta | T3 | Artery - Aorta | 314 | - | - | 0 |
| G2 | Artery - Coronary | T4 | Artery - Coronary | 182 | - | - | 0 |
| G2 | Artery - Tibial | T5 | Artery - Tibial | 464 | - | - | 0 |
| G3 | Thoracic Aorta | - | - | 0 | E3 | Thoracic Aorta | 3 |
| **G4** | Brain - Amygdala | T6 | Brain - Amygdala | 108 | - | - | 0 |
| **G4** | Brain - Anterior cingulate cortex (BA24) | T7 | Brain - Anterior cingulate cortex (BA24) | 134 | - | - | 0 |
| **G4** | Brain - Caudate (basal ganglia) | T8 | Brain - Caudate (basal ganglia) | 177 | - | - | 0 |
| **G4** | Brain - Cerebellar Hemisphere | T9 | Brain - Cerebellar Hemisphere | 154 | - | - | 0 |
| **G4** | Brain - Cerebellum | T10 | Brain - Cerebellum | 193 | E4 | Cerebellum | 2 |
| **G4** | Brain - Cortex | T11 | Brain - Cortex | 176 | - | - | 0 |
| **G4** | Brain - Frontal Cortex (BA9) | T12 | Brain - Frontal Cortex (BA9) | 145 | E5 | Frontal Cortex | 2 |
| **G4** | Brain - Hippocampus | T13 | Brain - Hippocampus | 135 | - | - | 0 |
| **G4** | Brain - Hypothalamus | T14 | Brain - Hypothalamus | 138 | - | - | 0 |
| **G4** | Brain - Nucleus accumbens (basal ganglia) | T15 | Brain - Nucleus accumbens (basal ganglia) | 161 | - | - | 0 |
| **G4** | Brain - Putamen (basal ganglia) | T16 | Brain - Putamen (basal ganglia) | 136 | - | - | 0 |
| **G4** | Brain - Spinal cord (cervical c-1) | T17 | Brain - Spinal cord (cervical c-1) | 102 | E6 | Spinal Cord | 2 |
| **G4** | Brain - Substantia nigra | T18 | Brain - Substantia nigra | 95 | - | - | 0 |
| **G4** | Occipital Lobe | - | - | 0 | E7 | Occipital Lobe | 2 |
| **G4** | Parietal Lobe | - | - | 0 | E8 | Parietal Lobe | 2 |
| **G4** | Temporal Lobe | - | - | 0 | E9 | Temporal Lobe | 2 |
| **G4** | Diencephalon | - | - | 0 | E10 | Diencephalon | 2 |
| **G5** | Breast - Mammary Tissue | T19 | Breast - Mammary Tissue | 306 | - | - | 0 |
| **G5** | Breast Epithelium | - | - | 0 | E11 | Breast Epithelium | 3 |
| G6 | Camera type eye | - | - | 0 | E12 | Camera type eye | 2 |
| **G7** | Colon - Sigmoid | T20 | Colon - Sigmoid | 254 | E13 | Sigmoid Colon | 4 |
| **G7** | Colon - Transverse | T21 | Colon - Transverse | 285 | E14 | Transverse Colon | 4 |
| **G8** | Esophagus - Gastroesophageal Junction | T22 | Esophagus - Gastroesophageal Junction | 263 | - | - | 0 |
| **G8** | Esophagus - Mucosa | T23 | Esophagus - Mucosa | 444 | - | - | 0 |
| **G8** | Esophagus - Muscularis | T24 | Esophagus - Muscularis | 394 | - | - | 0 |
| **G8** | Esophagus Muscularis Mucosa | - | - | 0 | E15 | Esophagus Muscularis Mucosa | 4 |
| **G8** | Esophagus Squamous Epithelium | - | - | 0 | E16 | Esophagus Squamous Epithelium | 4 |
| **G8** | Gastroesophageal Sphincter | - | - | 0 | E17 | Gastroesophageal Sphincter | 4 |
| **G8** | Stomach | T25 | Stomach | 272 | E18 | Stomach | 6 |
| **G9** | Heart | - | - | 0 | E19 | Heart | 3 |
| **G9** | Heart - Atrial Appendage | T26 | Heart - Atrial Appendage | 310 | - | - | 0 |
| **G9** | Heart - Left Ventricle | T27 | Heart - Left Ventricle | 352 | E20 | Heart - Left Ventricle | 2 |
| **G9** | Right Atrium Auricular Region | - | - | 0 | E21 | Right Atrium Auricular Region | 2 |
| **G10** | Adrenal Gland | T28 | Adrenal Gland | 205 | E22 | Adrenal Gland | 6 |
| **G10** | Kidney-Cortex | T29 | Kidney-Cortex | 50 | - | - | 0 |
| **G10** | Metanephros | - | - | 0 | E23 | Metanephros | 2 |
| **G11** | Liver | T30 | Liver | 188 | E24 | Liver | 4 |
| **G11** | Right Lobe of Liver | - | - | 0 | E25 | Right Lobe of Liver | 2 |
| **G12** | Lung | T31 | Lung | 474 | E26 | Lung | 2 |
| **G12** | Upper Lobe of Left Lung | - | - | 0 | E27 | Upper Lobe of Left Lung | 4 |
| G13 | Minor Salivary Gland | T32 | Minor Salivary Gland | 104 | - | - | 0 |
| **G14** | Muscle - Skeletal | T33 | Muscle - Skeletal | 625 | E28 | Skeletal Muscle Tissue | 2 |
| G15 | Gastrocnemius Medialis | - | - | 0 | E29 | Gastrocnemius Medialis | 5 |
| G16 | Tongue | - | - | 0 | E30 | Tongue | 2 |
| **G17** | Nerve - Tibial | T34 | Nerve - Tibial | 443 | E31 | Tibial Nerve | 4 |
| **G18** | Ovary | T35 | Ovary | 138 | E32 | Ovary | 2 |
| **G19** | Pancreas | T36 | Pancreas | 264 | E33 | Body of Pancreas | 2 |
| G20 | Peyer's Patch | - | - | 0 | E34 | Peyer's Patch | 4 |
| G21 | Pituitary | T37 | Pituitary | 191 | - | - | 0 |
| **G22** | Prostate | T38 | Prostate | 158 | - | - | 0 |
| **G22** | Prostate Gland | - | - | 0 | E35 | Prostate Gland | 2 |
| **G23** | Skin - Not Sun Exposed (Suprapubic) | T39 | Skin - Not Sun Exposed (Suprapubic) | 408 | E36 | Suprapubic Skin | 4 |
| **G23** | Skin - Sun Exposed (Lower leg) | T40 | Skin - Sun Exposed (Lower leg) | 520 | E37 | Lower Leg Skin | 4 |
| **G23** | Skin of Body | - | - | 0 | E38 | Skin of Body | 2 |
| G24 | Small Intestine - Terminal Ileum | T41 | Small Intestine - Terminal Ileum | 143 | - | - | 0 |
| **G25** | Spleen | T42 | Spleen | 169 | E39 | Spleen | 4 |
| **G26** | Testis | T43 | Testis | 280 | E40 | Testis | 2 |
| **G27** | Thyroid | T44 | Thyroid | 490 | - | - | 0 |
| **G27** | Thyroid Gland | - | - | 0 | E41 | Thyroid Gland | 8 |
| G28 | Umbilical Cord | - | - | 0 | E42 | Umbilical Cord | 2 |
| G29 | Urinary Bladder | - | - | 0 | E43 | Urinary Bladder | 2 |
| **G30** | Uterus | T45 | Uterus | 117 | E44 | Uterus | 4 |
| G31 | Vagina | T46 | Vagina | 124 | - | - | 0 |
| G32 | Whole Blood | T47 | Whole Blood | 460 | - | - | 0 |

*Tissue groups in bold are shared tissues in GTEx and ENCODE.
