# Supplementary figures and images for: TSEA-DB: a trait–tissue association map for human complex traits and diseases
Source: Nucleic Acids Res. 2019 Nov 4;48(D1):D1022–30. doi: 10.1093/nar/gkz957 (PMC7145616; doi:10.1093/nar/gkz957)

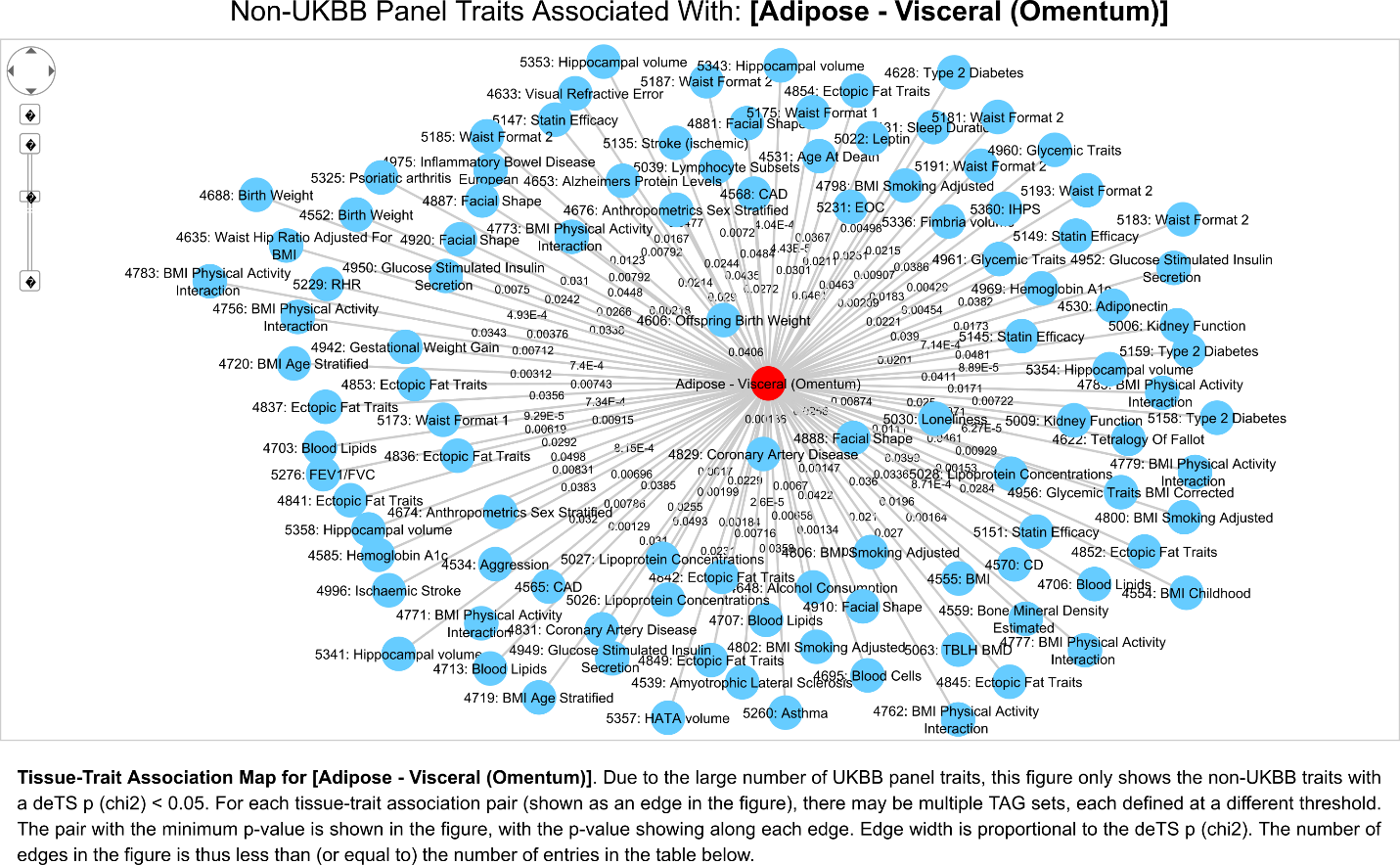


**Figure S1**. Illustration of the tissue page using Adipose – Visceral (Omentum) from GTEx.

Supplement: gkz957_Supplemental_Files [file gkz957_supplemental_files.zip › Figure S1.docx]
